# Circadian Disruptions Alter Consumption Timing and Exacerbate Binge-Like Eating in Mice

**DOI:** 10.5334/jcr.238

**Published:** 2025-04-22

**Authors:** Christopher J. Mancuso, Samantha P. Bedard, Lindsay Gillikin, P. Evelyna Kambanis, Emily Schmitt, Kyle P. De Young

**Affiliations:** 1 Department of Psychology, University of Wyoming, Laramie, WY, US; 2 Eating Disorders Clinical and Research Program, Massachusetts General Hospital, Boston, MA, US; 3 Department of Psychiatry, Harvard Medical School, Boston, MA, US; 4 Division of Kinesiology & Health, University of Wyoming, Laramie, WY, US; 5 Wyoming WWAMI Medical Education, Laramie, WY, US

**Keywords:** binge eating, circadian rhythms, rodent models, translational research

## Abstract

**Introduction:**

Circadian processes may be causal in the development and maintenance of binge eating. We used a rodent model of binge-like eating and a circadian disruption protocol to test whether circadian disruption causes increased consumption during 24h access to a high energy diet (HED).

**Method:**

Eight male mice underwent a two-week baseline with ad-lib standard chow and maintained a 12h light-dark schedule. Mice then completed two binge cycles. After, mice received a circadian disruption manipulation or remained on typical light schedule (i.e., were non-circadian disrupted). All mice received two binge cycles after manipulation. Chow and HED were measured every 12h and 24h. Independent samples *t*-tests compared consumption between the disrupted and non-disrupted groups.

**Results:**

Binge-like eating occurred in both experimental groups across all phases of the study. Circadian disrupted mice consumed more during HED access than non-disrupted mice, indicating that circadian disruptions may exacerbate binge-like eating. Circadian disruption also altered consumption timing; disrupted mice consumed more during typical rest hours (7:00–19:00) than non-disrupted mice but did not alter consumption during typical active hours (19:00–7:00).

**Conclusions:**

These results provide justification for research examining circadian processes implicated in binge eating. Future research may inform on the utility of circadian regulating adjunctive treatment (e.g., bright light therapy or exercise).

Binge eating is characterized by the consumption of an abnormally large amount of food over a discrete period (e.g., 1–2 hours) and is paired with a subjective loss of control over one’s eating [2]. Approximately 7.2% of US adults experience at least once-weekly binge-eating episodes [1]. Recurrent binge eating is associated with marked anxiety, depression, loneliness, and perceived lack of social support [7]. Individuals who experience binge eating carry elevated risk of Type 2 diabetes, hypertension, arthritis, and disrupted sleep [1]. Given the prevalence, pronounced psychological impairment, and associated health costs, explanatory models of binge eating are imperative to inform clinical practice.

Circadian processes may be causal in the development and maintenance of binge eating [24]. The Biobehavioral Circadian Model (BCM) outlines causal and moderating pathways of binge eating, positing that disruptions (e.g., shiftwork) to zeitgebers alter circadian regulation, which influences appetitive rhythms [10]. The BCM states that binge eating results from disrupted appetitive rhythms, such as irregular timing of consumption, particularly among those with eating disorder vulnerabilities [10]. Consistent with these propositions, bright light therapy, which synchronizes the circadian system, is effective in reducing binge eating [4]. While explanatory models generate novel questions on circadian pathways in binge eating, experimental work in humans is challenging [23].

Validated models of circadian disruption and binge eating in rodents can aid in isolating mechanisms outlined in circadian models of binge eating [21,8,9]. Though generalizability is limited [21], rodent models of binge eating reduce the influence of individual differences [15] and increase experimental control. Recent recommendations support the validity of binge eating models that provide weekly 24h access to highly palatable food that aligns with human behaviors [9]. As in humans, rodent feeding behavior is regulated by circadian processes that align with zeitgeber exposure [22], and binge-like eating protocols introduce palatable food at the start of the rodent active (dark) cycle [3,5]. Experimental manipulations of the environment mirroring shift work (e.g., reversal of light-dark cycle) are effective in desynchronizing circadian rhythms and inducing pathological changes [23,11,16]. An important direction for research on the role of circadian rhythms in binge eating is to combine rodent models of circadian disruption with a binge-like eating protocol [17].

To assess the impact of circadian disruption on binge-like eating, we combined a rodent model of binge-like eating that does not restrict consumption with a circadian disruption protocol using a 7h advance of lights every 24h for four weeks [12]. This study is the first to combine a circadian disruption with a binge-like eating paradigm, thus we elected to focus solely on behavioral outcomes and were interested in testing only for large effects to justify future research in this area with a broader scope. We hypothesized that circadian disruption would cause increased consumption during 24h access to a high energy diet. This would provide support for a causal role of circadian disruptions on exacerbations of binge-like eating, consistent with the BCM. We also sought to explore the impact of circadian disruption on circadian pattern of consumption. Mice are nocturnal, and consumption typically occurs during the active (dark) versus rest (light) phase [22]. Because the BCM posits appetitive disruptions occurring between circadian disruptions and binge eating, identifying changes to this consumption pattern would provide some support for broader appetitive disruptions.

## Method

### Animals

All procedures were approved by the Institutional Animal Care and Use Committee of the University of Wyoming and conducted in accordance with the American Association for the Accreditation of Laboratory Animal Care-approved guidelines and the Guide for Care and Use of Laboratory Animals [19]. Eight, 16-week-old male C57BL/6J (Jackson Labs, Bar Harbor, ME) mice were individually housed in an AAALAC-certified vivarium maintained at 18–21°C. All mice (*N* = 8) received ad libitum access to a standard rodent chow diet (Teklad no. 2014; 3.5% fat, ~11% sucrose, 3.2 kcal/g; Harlan Laboratories, Indianapolis, IN). During the baseline phase, mice were acclimated to their environment for two weeks and maintained on a 12h light-dark cycle (i.e., lights on at 7:00, lights off at 19:00). Mice did not have access to a running wheel to mitigate the potential effects of physical activity on consumption and circadian entrainment. To provide an enriched environment, nestlets and bedding were changed weekly.

### Binge-Like Eating Paradigm (Figure 1a)

**Figure 1:**
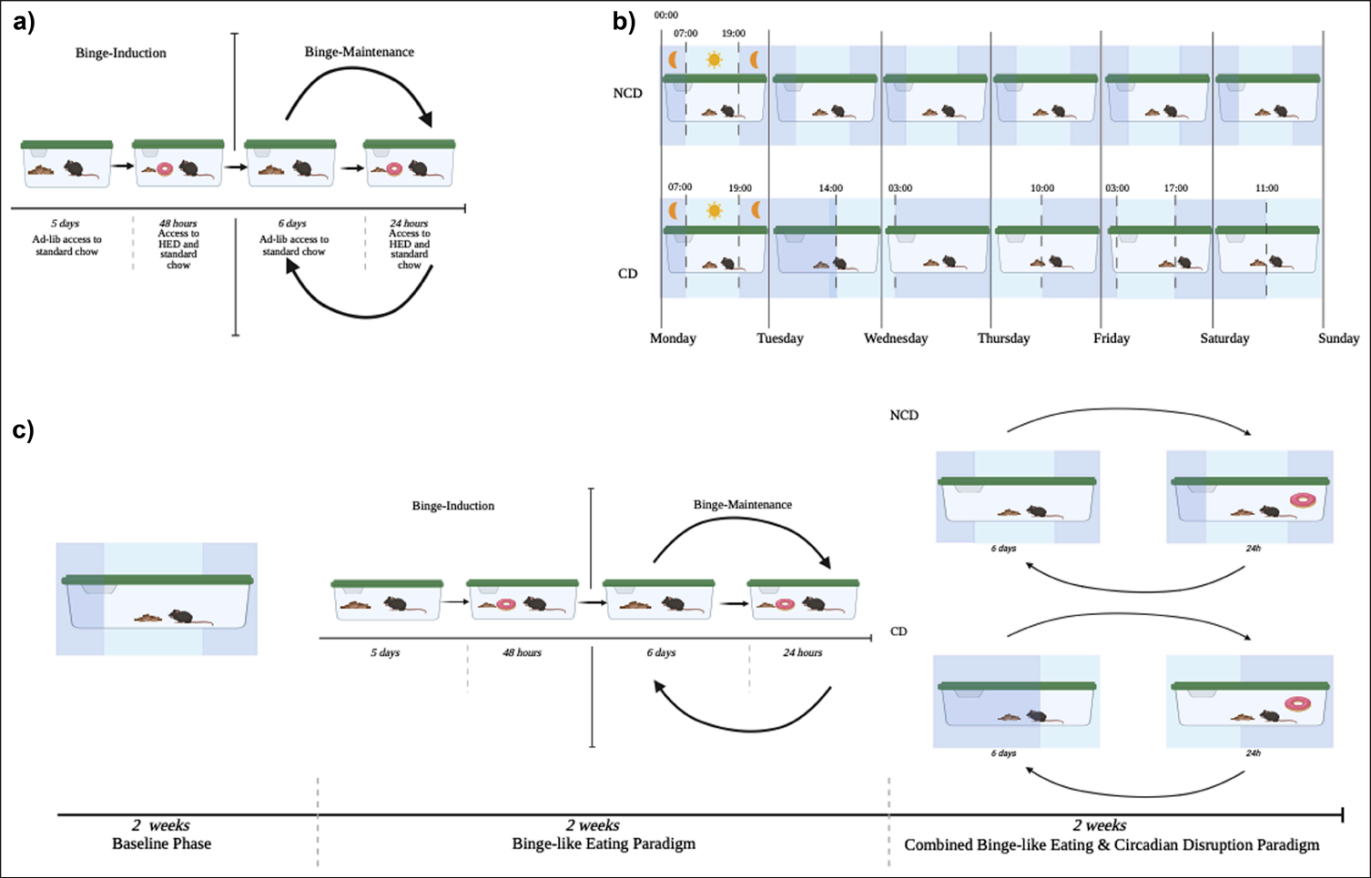
**a.** Binge-like Eating Paradiam. **b.** Circadian Disruption Paradigm. **c.** Study Timeline. *Note*. CD = Circadian Disrupted Mice, NCD = Non-circadian Disrupted Mice.

To induce binge-like eating, a validated protocol was applied [9]. In the binge-induction phase, mice received 48h access to a high energy diet (HED; Teklad no. 95217; 40% fat, ~16% sucrose, 4.3 kcal/g; Harlan Laboratories, Indianapolis, IN). After 48h access, HED was removed for five days, and only standard chow was available ad lib. This completed the induction. Next, 7-day binge cycles commenced; HED was introduced at lights off for 24h, and consumption of both standard chow and HED was measured 12h and 24h later. Following 24h ad lib HED access, HED was removed. Food was weighed manually every 12h via scale, to characterize dietary intake. Standard chow was stored in a hopper at the top of each cage. Diets (standard chow and HED) were provided separately within cages, allowing for accurate measurement of each food type.

### Circadian Disruption Paradigm (Figure 1b)

The applied paradigm elicits circadian rhythm desynchronization and induces behavioral (e.g., wheel-running activity) and pathophysiological alterations (e.g., hyperglycemia) in rodents [12,16]. All mice were initially exposed to the 12h light-dark cycle for the 2-week baseline and were maintained on this schedule during the 2-week binge-like eating paradigm. Then, mice randomized to circadian disruption underwent a progressive phase shift of 7h per day. On the first day, lights were turned on at 14:00 and off at 3:00. Each day for the next three weeks, the light schedule advanced 7h.

### General Procedure (Figure 1c)

During the 2-week baseline, mice had ad lib access to standard chow and were maintained on a 12h light-dark schedule. After baseline, mice completed two successive binge cycles^1^ over a two-week period. Then, mice were block randomized using a list randomizer: five mice received the circadian disruption, and three mice remained on the typical light schedule. All mice received two binge cycles^2^ following randomization to circadian condition. Standard chow and HED were weighed at 7:00 and 19:00 in the initial phase of the experiment. During circadian disruption, HED was introduced at lights off and measured 12h and 24h later. Mice were weighed once weekly, and measurements were conducted on the same scale throughout the duration of the experiment. Mice were handled minimally and were placed in a graduated cylinder during measurements. Mice did not have access to a running wheel to mitigate the potential effects of physical activity on consumption and circadian entrainment. To provide an enriched environment, nestlets and bedding were changed weekly.

### Statistical Analyses

Analyses were conducted using IBM Statistical Package for Social Sciences (SPSS Version 28; IBM, 2021). We sought to detect only large effects of circadian disruption on eating in a sample of mice [18]. Specifically, we adhered to the principle of reduction in that we used the minimum number of animals possible while sufficiently obtaining quality data. We analyzed consumption (kilocalories per gram of body weight; kcal/BW) for 24h intervals prior to, during, and following HED access consistent with comparable binge-like eating paradigms [9]. Independent samples *t*-tests compared consumption between the circadian disruption (CD; *n =* 5) and non-disruption (ND; *n =* 3) groups before and following the initiation of circadian disruption. Power analyses conducted using G*Power [13] indicated 80% power to detect Cohen’s *d =* 1.2. Paired sample *t*-tests compared consumption within experimental groups throughout the protocol. Power analyses indicated 80% power to detect an effect of (*d_z_*) 2.5 in the CD group. Repeated measures ANOVA compared weight wit hin experimental groups throughout the protocol, an exploratory analysis, therefore a priori power analyses were not conducted.

## Results

### Consumption During Binge-like Eating Paradigm (Table 1, Figure 2a)

**Table 1:** Dietary consumption during binge-like eating paradigm (kcal/BW).


	*M (SD)*	*M_DIFF_(SD)*	*T*(7)	*P*	*D_z_*

24h pre-HED	0.41 (0.07)				

24h HED	1.10 (0.17)	–0.69 (0.20)	–9.54	<.001	–3.37

24h HED	1.10 (0.17)				

24h post-HED	0.21 (0.06)	0.89 (0.17)	14.26	<.001	5.04

24h pre-HED	0.41 (0.07)				

24h post-HED	0.21 (0.06)	0.19 (0.08)	6.17	<.001	2.18


*Note*. Kcal/BW = kilocalories / body weight. 24h pre-HED = 24h prior to presentation of high energy diet. 24h HED = 24h presentation of high energy diet. 24h post-HED = 24h post-conclusion of high energy diet presentation. *M*(*SD*) = mean (standard deviation), *M_diff_* (*SD*) = mean difference (standard deviation). *d*= Cohen’s *d*.

**Figure 2:**
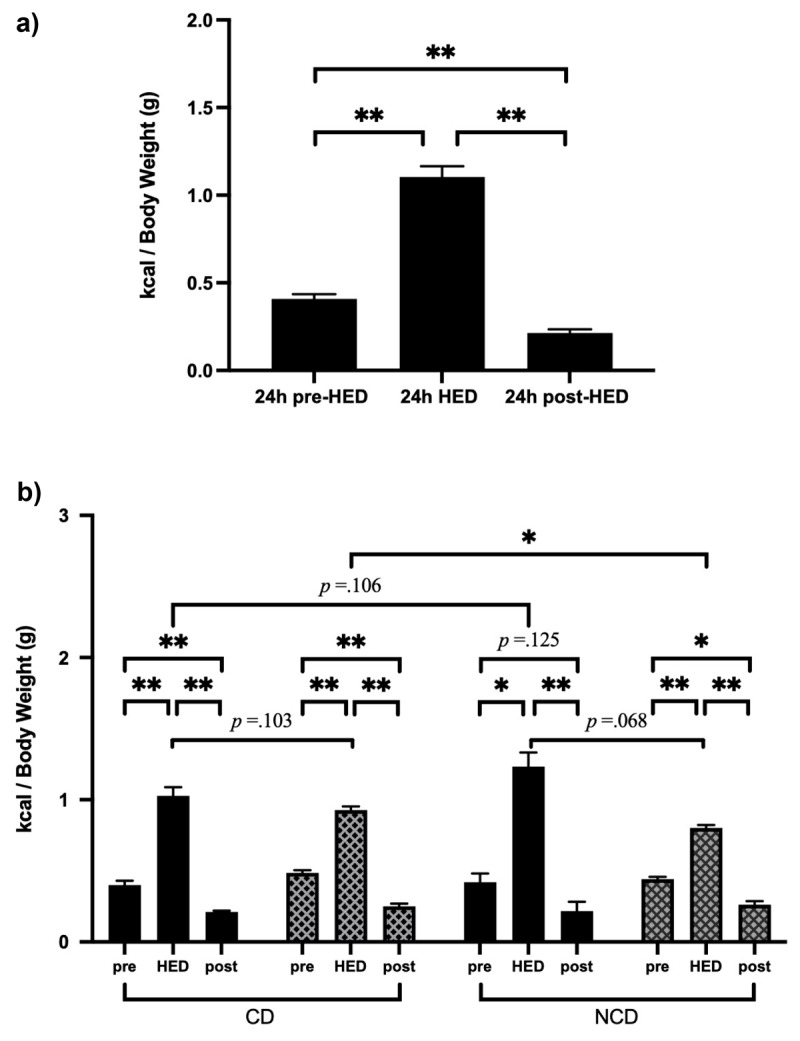
**a.** Dietary Consumption During Binge-like Eating Paradigm. **b.** Dietary Consumption During Combined Binge-like Eating and Circadian Disruption Paradiam. *Note*. CD = circadian disrupted Mice. NCD = non-circadian disrupted mice. HED = 24h presentation of high energy diet. pre = 24h pre-HED. post = 24h post-HED. *p < .05. **p < .01.

Comparison of consumption 24h before (standard chow only) and during HED access demonstrated that mice consumed over twice as many kcal/BW when provided access to HED. Further, consumption during the 24h period post-HED was 20% of consumption during HED access. Notably, consumption in the 24h period post-HED access was approximately half of consumption pre-HED access.

### Consumption During Combined Binge-like Eating and Circadian Disruption Paradigm (Table 2, Figure 2b)

**Table 2:** Dietary consumption during binge-like eating and circadian disruption paradigms (kcal/BW).


			*M_DIFF_*	*SD*	*T*(4)	*P*	*D_z_*

CD	*Binge-like Eating Paradigm*	24h pre-HED- 24h HED	–0.62	0.14	–9.35	<.001	–4.18

		24h HED- 24h post-HED	0.81	0.14	12.60	<.001	5.63

		24h pre-HED- 24h post-HED	0.18	0.06	6.41	.003	2.86

	*Combined Binge-like*	24h pre-HED- 24h HED	–0.44	0.07	–13.19	<.001	–5.90

	*Eating and Circadian*	24h HED – 24h post-HED	0.67	0.03	46.72	<.001	20.89

	*Disruption Paradigm*	24h pre-HED- 24h post-HED	0.23	0.05	9.76	<.001	4.36

NCD	*Binge-like Eating Paradigm*	24h pre-HED- 24h HED	–0.81	0.26	–5.24	.035	–3.02

		24h HED- 24h post-HED	1.01	0.17	10.15	.010	5.86

		24h pre-HED- 24h post-HED	0.20	0.13	2.55	.125	1.47

	*Combined Binge-like*	24h pre-HED- 24h HED	–0.35	0.04	–15.13	.004	–8.73

	*Eating and Circadian*	24h HED- 24h post-HED	0.53	0.07	12.13	.007	7.00

	*Disruption Paradigm*	24h pre-HED- 24h post-HED	0.18	0.04	6.78	.021	3.91


*Note*. Kcal/BW = kilocalories / body weight. CD = circadian disrupted mice. NCD = non-circadian disrupted mice. 24h pre-HED = 24h prior to presentation of high energy diet. 24h HED = 24h presentation of high energy diet. 24h post-HED = 24h post-conclusion of high energy diet presentation. *M_diff_* (*SD*) = mean difference (standard deviation). *d_z_*= Cohen’s *d*.

A similar consumption pattern was observed following randomization to CD or ND. That is, mice in *both* groups consumed more during 24h HED access relative to the 24h before and after access. As observed prior to circadian disruption, mice consumed significantly fewer kcal/BW in the 24h post- than the 24h pre-HED access.

### HED Consumption: Binge-like Eating Paradigm Versus Combined Binge-like Eating and Circadian Disruption Paradigm (Table 3)

**Table 3:** 24h HED consumption during binge-like eating versus combined binge-like eating and circadian disruption paradigms (kcal/BW).


	CONDITION	*M*	*SD*	*T*(7)	*P*	*D*

*Binge-like Eating Paradigm*	CD	1.02	0.13	–1.89	0.106	1.38

	NCD	1.23	0.17			

*Combined Binge-like* *Eating and Circadian Disruption Paradigm*	CD	0.92	0.059	3.21	0.018	2.35

	NCD	0.80	0.037			


*Note*. Kcal/BW = kilocalories / body weight. CD = circadian disrupted mice. NCD = non-circadian disrupted mice. HED = high energy diet. *d*= Cohen’s *d*.

Consumption of HED diet during 24h HED access in the binge-like eating paradigm did not differ between groups. However, during the combined binge-like eating and circadian disruption paradigm, CD mice consumed more of the HED diet during 24h HED access compared to ND mice.

### Timing of Dietary Consumption (Table 4)

**Table 4:** 12h HED and chow consumption during combined binge-like eating and circadian disruption paradigm (kcal/BW)


	CONDITION	*M*	*SD*	*T*(7)	*P*	*D*

19:00–7:00	CD	0.21	0.01	–0.88	0.465	–0.862

	NCD	0.25	0.07			

7:00–19:00	CD	0.15	0.02	4.42	0.003	3.22

	NCD	0.09	0.01			


*Note*. Kcal/BW = kilocalories / body weight. CD = circadian disrupted mice. NCD = non-circadian disrupted mice. HED = high energy diet. *d*= Cohen’s *d*.

Consumption during typical active hours (19:00–7:00) did not differ between groups during the binge-like eating paradigm. However, CD mice consumed significantly more than ND mice during typical rest hours (7:00–19:00) in the combined binge-like eating and circadian disruption paradigm.

### Weight

Weight change was not a focus of this study. However, given that weight is relevant to the understanding of disordered eating, we assessed weight change over the course of the study. Weight change was not observed over successive binge cycles in this study (*p* = 0.33) and reflects the findings detailed in comparable experimental designs (e.g., Murphy et al., 2018) [20]. In this limited-duration trial, and coupled with the observed restricted dietary intake following HED access, any effect of the experimental design on weight may have been negligible.

## Discussion

These findings support the idea that circadian disruption plays a critical role in exacerbating binge-like eating behaviors [10]. The observed increase in consumption during the light phase aligns with the Biobehavioral Circadian Model (BCM), which posits that disturbances in circadian rhythms can lead to alterations in appetitive controls [10]. Prior research suggests that circadian misalignment can disrupt metabolic processes and hunger signaling, further exacerbating disordered eating patterns [14,17]. The increase in total food intake under conditions of circadian disruption is consistent with previous studies demonstrating that such disruptions can lead to binge eating episodes [6,9,23]. Additionally, the alterations in feeding patterns observed in our study parallel findings in human populations, where irregular eating schedules are linked to increased risk of obesity and eating disorders [1,7]. Our results indicate that the typical nocturnal feeding behavior of rodents shifted towards increased consumption during the light phase. This shift not only highlights the impact of circadian misalignment on eating behaviors but also suggests a potential mechanism by which psychological stressors and environmental cues may contribute to binge eating disorders [24].

Future research should investigate the underlying neurobiological mechanisms that contribute to these circadian disruptions and their impact on binge eating. Understanding these mechanisms may illuminate new targets for therapeutic interventions aimed at restoring proper circadian regulation [17,10,4]. Rodent models, as utilized in this study, can provide further insights into the complex interactions between circadian rhythms and eating behaviors, offering a pathway for developing effective treatment strategies [21]. These results provide justification for future research examining circadian processes implicated in binge eating. Future research on this topic may inform on the potential utility of circadian regulation as an augment to treatment (e.g., bright light therapy) and on the phenomenology of eating among shift workers. Basic research is vital for identifying potentially modifiable mechanisms to reduce binge eating.

## Statement of Human and Animal Rights

All experimental procedures involving animals were conducted in accordance with the All procedures were approved by the Institutional Animal Care and Use Committee of the University of Wyoming.

## Footnotes

A one-week binge-induction (48h HED access, 5 days ad-lib chow only) followed by one week of binge-maintenance (24h HED access, 6 days ad-lib chow only).

Two weeks of binge-maintenance (24h HED access, 6 days ad-lib chow only).
